# Primary Thyroid Lymphoma: External Beam Radiation Therapy Induced Thyroiditis Mimics Residual Disease on Serial 18F-FDG PET/CT Imaging

**DOI:** 10.4274/mirt.07088

**Published:** 2018-02-01

**Authors:** William Makis, Anthony Ciarallo, Stephan Probst

**Affiliations:** 1 Cross Cancer Institute, Department of Diagnostic Imaging, Edmonton, Canada; 2 McGill University Health Centre, Department of Nuclear Medicine, Montreal, Canada; 3 Jewish General Hospital, Department of Nuclear Medicine, Montreal, Canada

**Keywords:** Thyroid lymphoma, thyroiditis, pitfall, artifact, ^18^F-FDG, PET

## Abstract

A 67-year-old female patient with no prior history of benign thyroid disease was diagnosed with primary thyroid lymphoma and was staged with^18^F-fluoro-2-deoxy-D-glucose (^18^F-FDG) positron emission tomography/computed tomography (PET/CT). She was treated with chemotherapy and external beam radiation therapy, and a follow-up PET/CT showed significant reduction in the size of the thyroid lymphoma with persistent intense ^18^F-FDG uptake, which was interpreted as partial response to therapy. However, two subsequent PET/CT studies showed no change in the persistent intense ^18^F-FDG uptake in the thyroid and a biopsy confirmed the presence of thyroiditis with no evidence of residual lymphoma. Follow-up PET/CTs performed over the subsequent three years showed stable intensely ^18^F-FDG avid thyroiditis with no evidence of lymphoma recurrence. We present the imaging characteristics of a long term radiation treatment induced thyroiditis mimicking ^18^F-FDG avid residual disease on PET/CT.

## Abstract (tr)

İyi huylu tiroid hastalığı anamnezi olmayan 67 yaşında bir kadına primer tiroid lenfoması tanısı konularak ^18^F-florodeoksiglukoz (^18^F-FDG) pozitron emisyon tomografisi/bilgisayarlı tomografi (PET/BT) ile evreleme yapılmıştı. Kemoterapi ve eksternal radyasyon tedavisi uygulanması sonrası takip PET/BT’de tiroid lenfomasında anlamlı çap küçülmesi saptanarak, devam eden yoğun ^18^F-FDG tutulumu tedaviye kısmi cevap olarak değerlendirilmişti. Ne var ki, takip eden iki PET/BT’de tiroiddeki persistan yoğun ^18^F-FDG tutulumu gerilememiş ve biyopside rezidü lenfoma olmaksızın tiroidit bulunmuştu. Takip eden üç yıl boyunca çekilen PET/BT’lerde stabil yoğun ^18^F-FDG tutan tiroidit, lenfoma nüksü olmaksızın devam etmiştir. Burada PET/BT’de ^18^F-FDG tutan rezidüel hastalığı taklit eden uzun dönemli radyasyona bağlı tiroidite bağlı görüntüleme özelliklerini sunuyoruz.

## Figures and Tables

**Figure 1:**
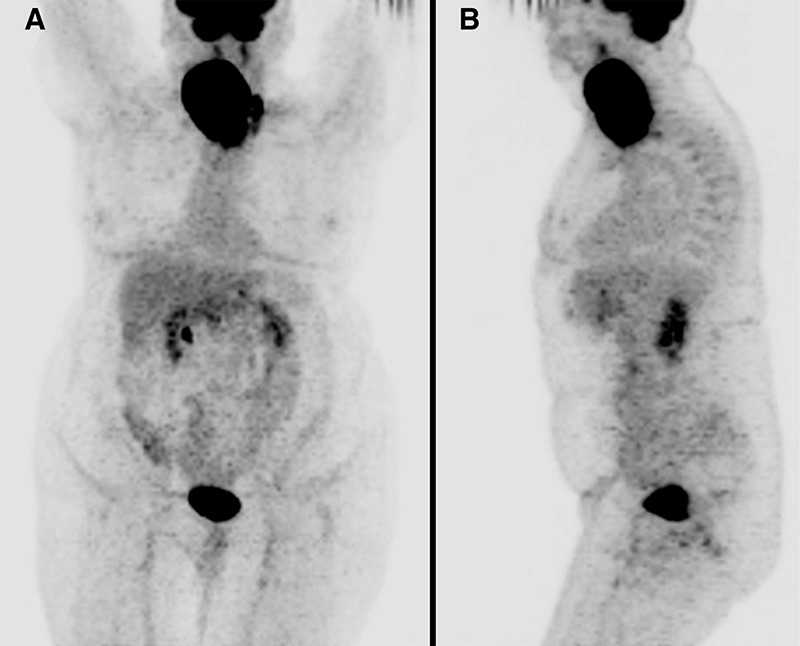
A 67-year-old woman with no prior history of benign thyroid disease presented with a two month history of fevers, night sweats, and rapidly enlarging neck mass. Biopsy of the right thyroid lobe revealed a diffuse large B-cell lymphoma (DLBCL). A staging ^18^F-fluoro-2-deoxy-D-glucose (^18^F-FDG) positron emission tomography/computed tomography (PET/CT) (Discovery-ST, GE Healthcare, WI, USA) was performed and maximum intensity projection (MIP) images, (A) anterior and (B) left lateral, showed a large intensely ^18^F-FDG avid mass in the right neck with no distant metastases.

**Figure 2:**
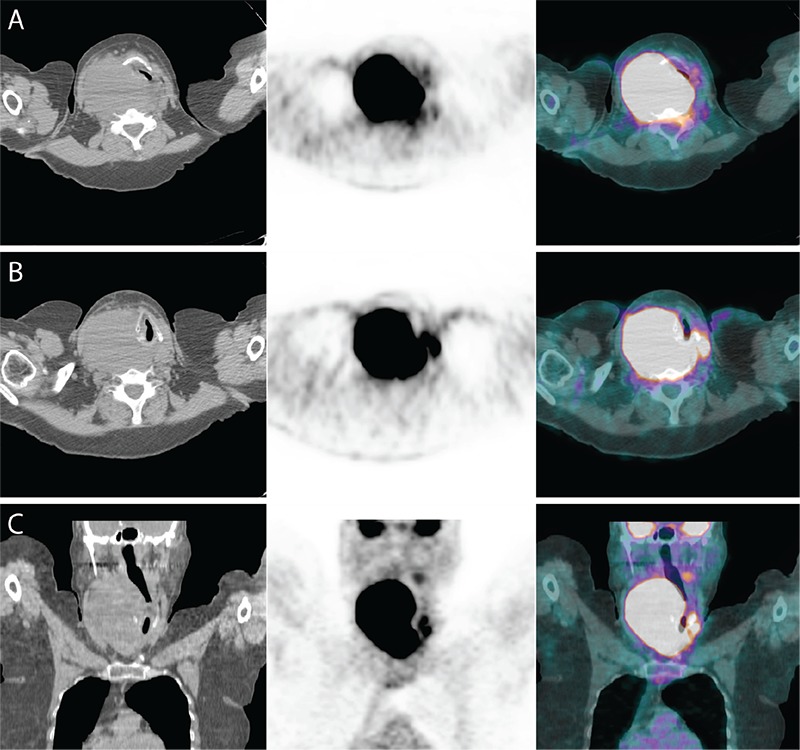
(A, B) Transaxial and (C) coronal views of the neck with CT (left), PET (middle), and PET/CT fusion (right) images show right thyroid DLBCL, measuring 13x9 cm with maximum standardized uptake value (SUV_max_) 54, compressing the trachea to a narrow slit left of the midline.

**Figure 3:**
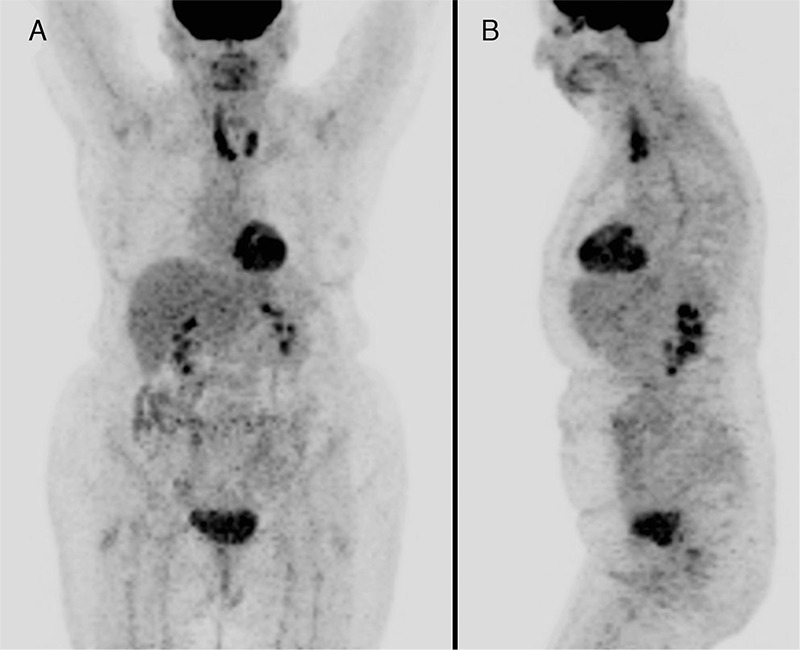
The patient was treated with 3 cycles of CHOP chemotherapy (cyclophosphamide, doxorubicin, vincristine, prednisone) followed by external beam radiation therapy. The first follow-up PET/CT was performed 3 months after the end of radiation therapy and MIP views, (A) anterior and (B) left lateral, show a dramatic reduction in the size of right thyroid DLBCL and resolution of tracheal compression with persistently intense ^18^F-FDG uptake in both thyroid lobes with SUV_max_ 8.9 on the right. These findings were interpreted as partial response to therapy, however since the patient was asymptomatic, a conservative management approach was taken.

**Figure 4:**
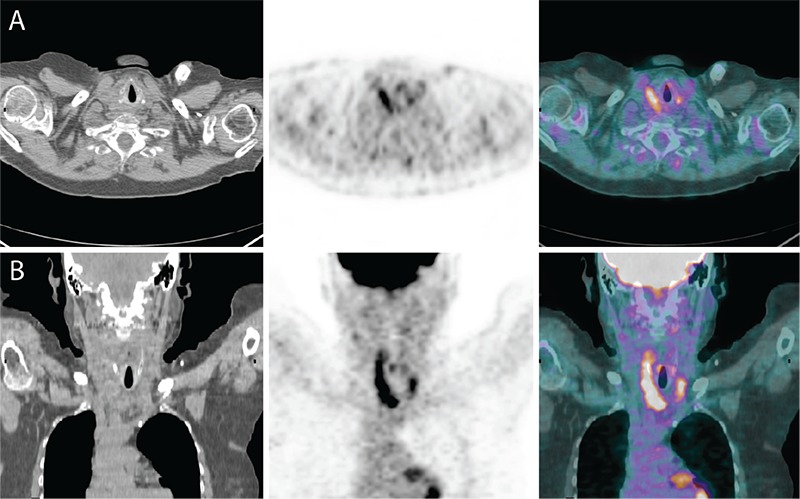
(A) Transaxial and (B) coronal views of the post therapy PET/CT showing apparent partial response to therapy.

**Figure 5:**
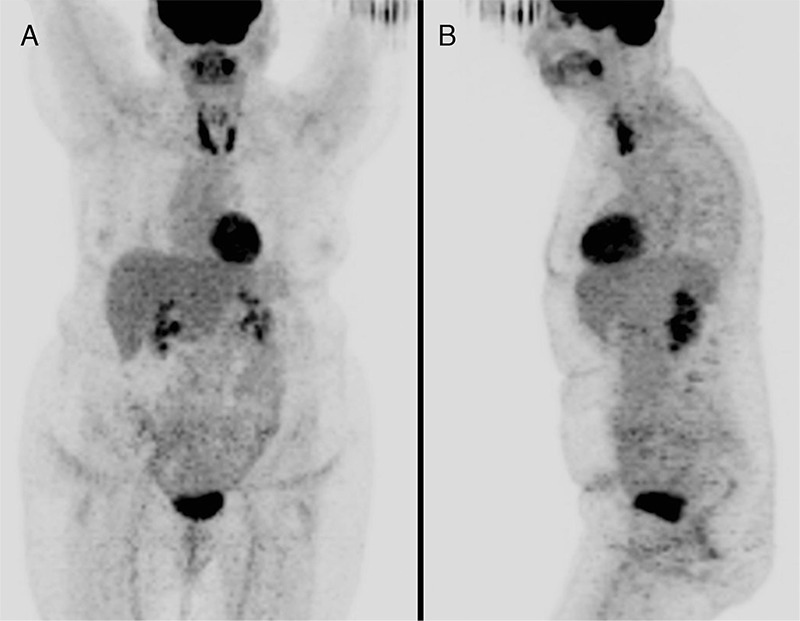
A second follow-up PET/CT was performed one year later. SUVmax of right thyroid lobe was 10.2, unchanged according to PET Response Criteria in Solid Tumors (PERCIST 1.0). The pattern of 18F-FDG uptake and distribution in the thyroid gland were also unchanged. Biopsy of both thyroid lobes revealed thyroiditis with no evidence of residual lymphoma. A third follow-up PET/CT was performed 2 years later and SUVmax in the right thyroid lobe was 9.5 (unchanged according to PERCIST) with stable pattern of 18F-FDG uptake and distribution, consistent with remission. Primary thyroid lymphoma makes up 1-5% of thyroid malignancies and less than 2% of extranodal lymphomas (1). It is more common in women, and patients present in the seventh decade of life with rapidly enlarging neck mass (1,2,3,4). The use of 18F-FDG PET/CT to stage primary thyroid lymphoma and evaluate response to therapy has been previously reported in the literature (5,6). Recent studies have raised concern about high rate of false positives on PET/CT performed following therapy for thyroid lymphoma, although these have been attributed to Hashimoto’s thyroiditis (7,8,9,10,11,12). External beam radiation therapy can produce both inflammatory and proliferative changes in the thyroid gland with increases in macrophage populations (9,10). Studies have shown that ^18^F-FDG uptake is increased in head and neck soft tissues affected by such inflammatory and proliferative processes and lasts several months after the completion of radiation therapy (11,12,10). The present case is an example of a long-term post-treatment thyroiditis, which remained intensely ^18^F-FDG avid but stable on several follow-up PET/CTs spanning three years, which to our knowledge has not been previously reported in the literature.
